# Effects of Dietary Fat Intake on HDL Metabolism

**DOI:** 10.14740/jocmr2030w

**Published:** 2014-12-29

**Authors:** Hidekatsu Yanai, Hisayuki Katsuyama, Hidetaka Hamasaki, Shinichi Abe, Norio Tada, Akahito Sako

**Affiliations:** a Department of Internal Medicine, National Center for Global Health and Medicine, Kohnodai Hospital, Chiba, Japan; b The Jikei University School of Medicine, Tokyo, Japan

**Keywords:** Coronary heart diseases, Fatty acids, Fish oils, High-density lipoprotein, Plant sterols

## Abstract

High-density lipoprotein (HDL) is a lipoprotein which has anti-atherogenic property by reversing cholesterol transport from the peripheral tissues to liver. Low HDL-cholesterol (HDL-C) as well as high low-density lipoprotein-cholesterol (LDL-C) is associated with the development of coronary heart diseases (CHD). Various epidemiological studies have suggested that the development of CHD increase in individuals with less than 40 mg/dL of HDL-C. In spite of accumulation of evidences suggesting a significant association between low HDL-C and CHD, effects of dietary factors on HDL metabolism remained largely unknown. We reviewed published articles about effects of dietary fat intake on HDL metabolism. The substitution of fatty acids (FA) for carbohydrates is beneficially associated with HDL metabolism. Monounsaturated FA intake may not affect HDL-C. Trans-FA is significantly associated with reduction of HDL-C, and is also adversely related with total cholesterol/HDL-C. Fish oils consumption, especially docosahexaenoic acid consumption, may be favorably associated with HDL metabolism. Although plant sterols and stanols may not affect HDL-C, policosanol intake is associated with a clinically significant decrease in the LDL/HDL ratio.

## Introduction

Atherogenic dyslipidemia is characterized as elevated serum levels of triglyceride (TG) and low-density lipoprotein-cholesterol (LDL-C), and low serum levels of high-density lipoprotein-cholesterol (HDL-C). Since HDL is an anti-atherogenic lipoprotein which plays a role in reversing cholesterol transport from the peripheral tissues to the liver, low HDL-C level induces the development of coronary heart diseases (CHD) [1, 2], and cerebrovascular diseases (CVD) [3].

A significant influence of low HDL-C on CHD and CVD has been widely known; however, roles of dietary factors for HDL metabolism remained largely unknown. We reviewed published articles about effects of dietary factors, especially dietary fat intake on HDL metabolism. We regarded systematic reviews and meta-analyses as important articles in this review.

## Effects of Various Fatty Acids (FA) Intake on HDL Metabolism

Mensink et al calculated the effect of changes in carbohydrate and FA intake on serum lipids, reviewing 27 controlled trials published between 1970 and 1991 (Table 1) [4-8]. They found that all types of FA ingestion elevated HDL-C when substituted for carbohydrates, but the effect decreased with increasing unsaturation of FA. Another meta-analysis was performed to examine the relationship between milk fat containing dairy foods and cardiovascular risk factors [5]. This study indicated that a diet higher in saturated FA (SFA) from whole milk and butter increases HDL-C when substituted for carbohydrates or unsaturated FA (USFA). Results from these two studies indicate that the substitution of FA for carbohydrates is beneficially associated with HDL metabolism, and also suggest that SFA is favorably associated with HDL-C. Mensink et al performed the meta-analysis of 60 selected trials and calculated the effects of the amount and type of fat on the total cholesterol (TC)/HDL-C [6]. TC/HDL did not change if carbohydrates replaced SFA, but it decreased if cis-USFA replaced SFA. The effect on TC/HDL of replacing trans-FA (TFA) with a mix of carbohydrates and cis-USFA was almost twice as large as that of replacing SFA. Briefly, TC/HDL-C was reduced by the replacement of TFA with SFA by 0.019; the replacement with cis-monounsaturated FA (MUFA) by 0.048; and the replacement with cis-polyunsaturated FA (PUFA) by 0.054. Coronary risk was reduced most effectively when TFA and SFA were replaced with cis-USFA.

**Table 1:** Meta-Analyses for Effects of Various FA Intake on HDL Metabolism

Authors	Study design	Subjects	Results/conclusions
Mensink et al [4]	Effects of changes in carbohydrate and FA intake on serum lipids	Twenty-seven controlled trials	All FA elevated HDL-C when substituted for carbohydrates
Huth et al [5]	The relationship between milk fat containing dairy foods and cardiovascular health	The published research including observational studies and short-term intervention studies, and reviews	A diet higher in SFA from whole milk and butter increases HDL-C when substituted for carbohydrates or USFA
Mensink et al [6]	Effects of the amount and type of fat on TC/HDL-C and on other lipids	Sixty controlled trials	Replacement of carbohydrates with SFA did not change TC/HDL-C, but replacement with cis-USFA decreased. Replacement of TFA with SFA decreased TC/HDL-C by 0.019; with cis-MUFA, by 0.048; and with cis-PUFA, by 0.054
Salas-Salvado et al [7]	Effects of CLA on metabolic parameters	Healthy humans or patients with overweight, obesity, metabolic syndrome, or diabetes	CLA isomers decreases HDL-C
Wendland et al [8]	Effects of dietary supplementation with ALA on cardiovascular risk markers	Fourteen studies with minimum treatment duration of 4 weeks	There was a small but clinically unimportant decrease in HDL (0.39 mg/dL, 95% CI: -0.77 - 0.00, P < 0.01)

ALA: alpha linolenic acid; CI: confidence interval; CLA: conjugated linoleic acid; FA: fatty acids; HDL-C: high-density lipoprotein- cholesterol; SFA: saturated fatty acids; USFA: unsaturated fatty acids; TC: total cholesterol; TFA: trans-fatty acids.

Salas-Salvado et al reviewed the clinical trials on humans that evaluate how mixtures of conjugated linoleic acid (CLA) isomers administered as supplements or CLA-enriched products can affect plasma lipids, and they found that some of studies have observed that various CLA isomers decreases HDL-C [7]. Wendland et al performed systematic review and meta-analysis to determine whether dietary supplementation with alpha linolenic acid (ALA) can modify coronary risk factors [8]. There was a small but clinically non-significant decrease in HDL-C due to ALA ingestion.

Schwingshackl et al analyzed long-term, randomized controlled trials (RCTs) to investigate the effects of MUFA on cardiovascular risk factors (Table 2) [9-11]. Dietary regimens with a high amount of MUFA (> 12%) were compared to those with ≤ 12%. A total of 12 studies met the inclusion criteria. HDL-C was not significantly affected by the percentage of MUFA.

**Table 2:** Meta-Analyses for the Effects of MUFA and TFA on HDL Metabolism

Authors	Study design	Subjects	Results/conclusions
Schwingshackl et al [9]	Effects of MUFA on cardiovascular risk factors. Dietary regimens with a high amount of MUFA (> 12%) were compared to those with ≤ 12%.	Twelve studies	No effect on HDL-C
Mozaffarian et al [10]	Effects of TFA consumption on CHD	Medline publications examining TFA consumption and CHD risk factors or outcomes in humans	The effects of TFA consumption on risk factors most consistently seen in both controlled trials and observational studies included reduction of HDL-C
Mozaffarian et al [11]	Quantitative estimates of CHD effects if a person's PHVO consumption were to be replaced with alternative fats and oils based on randomized dietary trials and prospective observational studies	Meta-analyses of the effects of TFAs on blood lipids and lipoproteins in controlled dietary trials and associations of habitual TFA consumption with CHD outcomes in prospective cohort studies	In controlled trials, each 1% energy replacement of TFA with SFA, MUFA and PUFA, respectively, decreased the TC/HDL-C by 0.31, 0.54 and 0.67, respectively

CHD: coronary heart diseases; MUFA: monounsaturated fatty acids; HDL-C: high-density lipoprotein-cholesterol; PHVO: partially hydrogenated vegetable oils; PUFA: polyunsaturated fatty acids, SFA: saturated fatty acids; TC: total cholesterol.

Recently, TFA has been reported to adversely affect cardiovascular health, and Mozaffarian et al reviewed the evidence for effects of TFA consumption on CHD risks. They found that reduction of HDL-C by TFA consumption was observed in both controlled trials and observational studies [10]. These effects were most prominent in comparison with cis-USFA, and adverse effect of TFA on TC/HDL-C was also found in comparison with SFA. Mozaffarian et al performed a meta-analysis of the effects of TFA on serum lipids in controlled dietary trials. In controlled trials, each 1% energy replacement of TFA with SFA, MUFA and PUFA decreased TC/HDL-C by 0.31, 0.54 and 0.67, respectively [11].

## Effects of Fish Oils Intake on HDL Metabolism

Fish oils have been widely reported to be a useful supplement to reduce serum TG levels in individuals with hyperlipidemia; however, effects of fish oils on serum HDL-C remained obscure. Lewis et al reviewed all RCTs from 1994 to 2003 which addressed the efficacy of long-chain omega-3 FA for dyslipidemia, and they found that 10 studies reported long-chain omega-3 FA to be effective in the treatment of hypertriglyceridemia (Table 3) [12-16]. According to accumulation of the data obtained from 10 studies, the average increase in HDL-C was 10%. Eslick et al performed the meta-analysis to quantitatively evaluate all RCTs of fish oils in hyperlipidemic individuals [13]. Their final analysis comprised of 47 studies showed that taking fish oils (daily intake of 3.25 g of eicosapentaenoic acid (EPA) and/or docosahexaenoic acid (DHA)) produced a modest increase in HDL-C (0.39 mg/dL, 95% CI: 0.00 - 0.77) which was not statistically significant. Pei et al performed a systematic review and meta-analysis of the effect of n-3 PUFA consumption on plasma lipids in patients with end-stage renal disease [14]. They reviewed evidences obtained from 10 RCTs including 557 patients with end-stage renal disease. The pooled analysis revealed that n-3 PUFA intake elevated HDL-C by 9.67 mg/dL, which was not statistically significant. Certain algae contain the n-3 FA, DHA. Bernstein et al examined the relationship between algal oil supplementation and CHD risk factors, by conducting a systematic review of RCTs published between 1996 and 2011 [15]. They identified 11 RCTs with 485 healthy participants, and the median dose of algal DHA was 1.68 g/day. The pooled estimate for the change in HDL-C was 2.71 mg/dL (95% CI: 1.93 - 3.87), suggesting that DHA supplementation from algal oil increases HDL-C in healthy individuals. Wei et al performed a meta-analysis of RCTs of monotherapy with EPA (n = 10), DHA (n = 17), or EPA vs. DHA (n = 6) [16]. Compared with placebo, DHA raised HDL-C (4.49 mg/dL; 95% CI: 3.50 - 5.48), whereas EPA did not raise HDL-C.

**Table 3:** Meta-Analyses for Effects of Fish Oils Intake on HDL Metabolism

Authors	Study design	Subjects	Results/conclusions
Lewis et al [12]	Efficacy of long-chain omega-3 FA as secondary agents for prevention of hypertriglyceridemia	Ten studies	Average increase in HDL was 10%
Eslick et al [13]	Effects of fish oils on serum lipids in hyperlipidemic subjects	Forty-seven studies, subjects taking fish oils (daily intake of 3.25 g of EPA and/or DHA)	Taking fish oils produced very slight increases in HDL (0.39 mg/dL, 95% CI: 0.00 - 0.77)
Pei et al [14]	Effect of n-3 PUFA consumption on plasma lipids	Five hundred fifty-seven patients with end-stage renal disease	Consumption of n-3 PUFA elevated HDL-C by 9.67 mg/dL, but these changes were not statistically significant
Bernstein et al [15]	Certain algae contain the DHA. The relation between algal oil supplementation and cardiovascular disease risk factors	Eleven randomized controlled trials with 485 healthy participants	The pooled estimate for the change in HDL-C was 2.71 mg/dL (95% CI: 1.93 - 3.87)
Wei et al [16]	Effects of EPA and DHA on serum lipids	Monotherapy with EPA (n = 10), DHA (n = 17), or EPA vs. DHA (n = 6)	DHA raised HDL (4.49 mg/dL; 95% CI: 3.50 - 5.48) compared with placebo, whereas EPA did not

CI: confidence interval; DHA: docosahexaenoic acid; EPA: eicosapentaenoic acid; FA: fatty acids; HDL-C: high-density lipoprotein-cholesterol; PUFA: polyunsaturated fatty acids.

## Effects of Plant Sterols and Stanols on HDL Metabolism

Plant sterols and stanols have a similar chemical structure and function to cholesterol. Therefore, plant sterols and stanols ingestion are sometimes suggested to beneficially modify serum lipids. Plant sterols have a higher degree of absorption as compared with plant stanols. Talati et al performed a meta-analysis of RCTs to compare the effect of plant sterols with plant stanols on serum lipid levels in healthy individuals and also patients with hypercholesterolemia (Table 4) [17-20]. Fourteen studies (n = 531) met the inclusion criteria. There was no statistically or clinically significant difference between plant sterols and plant stanols in their abilities to modify HDL-C. Seppo et al conducted a meta-analysis of four RCTs to evaluate the effect of low-fat milk products enriched with plant stanol esters on serum lipids [18]. Each stanol-ester-enriched milk product provided daily 2 g of stanols, and the intervention period was 5 weeks. A total of 199 hypercholesterolemic subjects were included. There were no significant differences between the placebo group and the stanol group in pooled HDL-C. Moruisi et al conducted a systematic review that investigates the efficacy of plant sterols and stanols in modifying serum lipids in subjects with familial hypercholesterolemia (FH) [19]. The subjects studied were heterozygous FH subjects, aged 2 - 69 years old with baseline TC and LDL-C concentrations of 270.7 mg/dL and 208.8 mg/dL, respectively. Result showed that plant sterols and stanols did not affect HDL-C. Policosanol is a very long chain aliphatic alcohol derived from the wax constituent of plants [21]. The original policosanol supplement has been approved as a cholesterol-lowering drug in over 25 countries. Chen et al performed systematic review and meta-analysis of RCTs, to compare the efficacy and safety of plant sterols and stanols as well as policosanol in the treatment of CHD [20]. A total of 4,596 patients from 52 eligible studies were included for their analysis. Policosanol affected HDL-C more favorably than plant sterols and stanols. Policosanol induced a clinically significant decrease in the LDL/HDL ratio.

**Table 4:** Meta-Analyses for Effects of Plant Sterols and Stanols on HDL Metabolism

Authors	Study design	Subjects	Results/conclusions
Talati et al [17]	Comparison between the effect of plant sterols vs. plant stanols on serum lipids	Healthy subjects or patients with hypercholesterolemia, 14 studies (n = 531)	No statistically or clinically significant difference between plant sterols and plant stanols in their abilities to modify HDL-C
Seppo et al [18]	Effects of ingestion of low-fat milk products enriched with plant stanol esters (2 g/day) for 5 weeks on serum lipids	A total of 199 hypercholesterolemic subjects	There were no significant differences between the groups in pooled HDL-C
Moruisi et al [19]	Efficacy of plant sterols/stanols for 4 weeks to 3 months in lowering TC and LDL-C in FH subjects	Heterozygous FH patients, aged 2 - 69 years old	HDL-C were not affected
Chen et al [20]	Comparison of the efficacy and safety of plant sterols and stanols with policosanol in serum lipids	A total of 4,596 patients from 52 eligible studies	Policosanol affected HDL-C more favorably than plant sterols and stanols

FH: familial hypercholesterolemia; HDL-C: high-density lipoprotein-cholesterol; LDL-C: low-density lipoprotein-cholesterol; TC: total cholesterol.

## Conclusion

The substitution of FA for carbohydrates is beneficially associated with HDL metabolism. MUFA intake may not affect HDL-C. TFA is significantly associated with reduction of HDL-C, and is also adversely related with TC/HDL-C, coronary risks. Fish oils consumption, especially DHA consumption, may be favorably associated with HDL metabolism. Although plant sterols and stanols may not affect HDL-C, policosanol intake is associated with a clinically significant decrease in the LDL/HDL ratio.
